# Supplementary material for: PI3Kδ activates E2F1 synthesis in response to mRNA translation stress
Source: Nat Commun. 2017 Dec 13;8:2103. doi: 10.1038/s41467-017-02282-w (PMC5727396; doi:10.1038/s41467-017-02282-w)
Supplement: Supplementary file 1 — Supplementary Information [file 41467_2017_2282_MOESM1_ESM.pdf]

Supplementary Figure 1

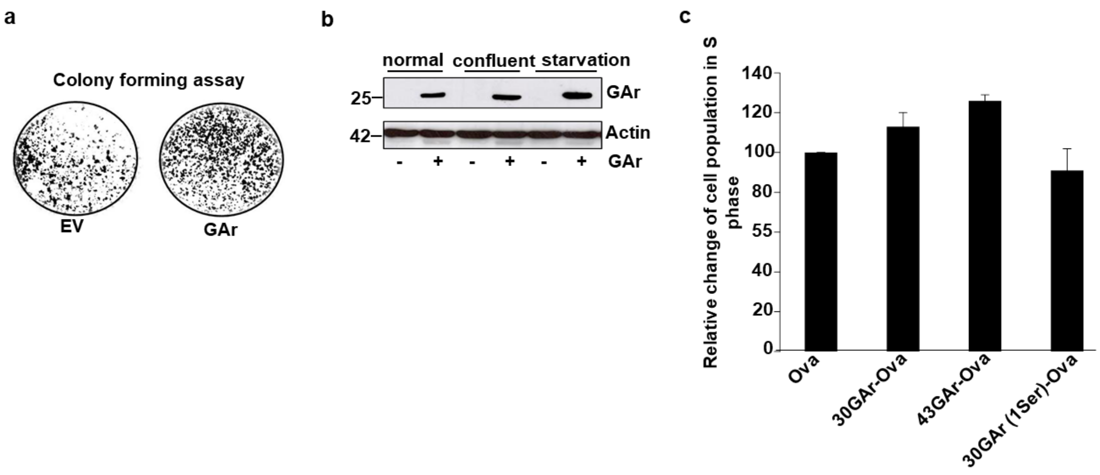

Supplementary Figure 1. a) Colonies of H1299 cells expressing the gly-ala repeat (GAr) of the Epstein Barr virus-encoded EBNA1, or plasmid empty vector (EV) after 15 days in selection medium. b) The western blot shows GAr expression levels under indicated growth conditions. c) Flow cytometry analysis of H1299 cells expressing various GAr-sequences fused to 5' of the Ovalbumin (Ova) open reading frame. The length of the GAr determines its translation inhibitory capacity and the longer the more efficient. The 30GAr (1Ser) has a serine residue inserted in a 30 amino acid long GAr sequence that disrupts the effect of the GAr on mRNA translation suppression [1]. The values represent the mean data from three independent experiments with s.d. Data relates to Figure 1.

Supplementary Figure 2

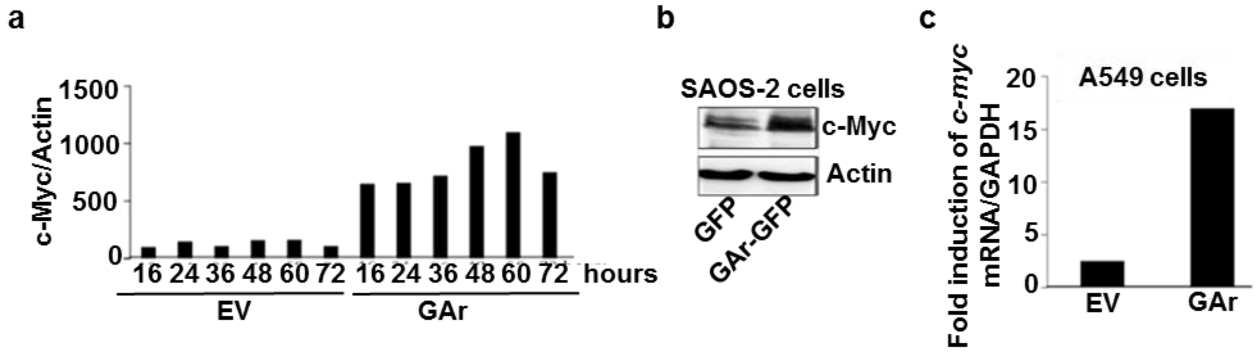

Supplementary Figure 2. a) Time course on c-Myc expression levels in SAOS-2 cells expressing GAr compared to control (EV) normalized with actin. b) Induction of c-Myc by the GAr fused to GFP in SAOS-2 cells. c) Induction of *c-myc* mRNA levels by the GAr in A549 cells. Data relates to figure 1.

Supplementary Figure 3

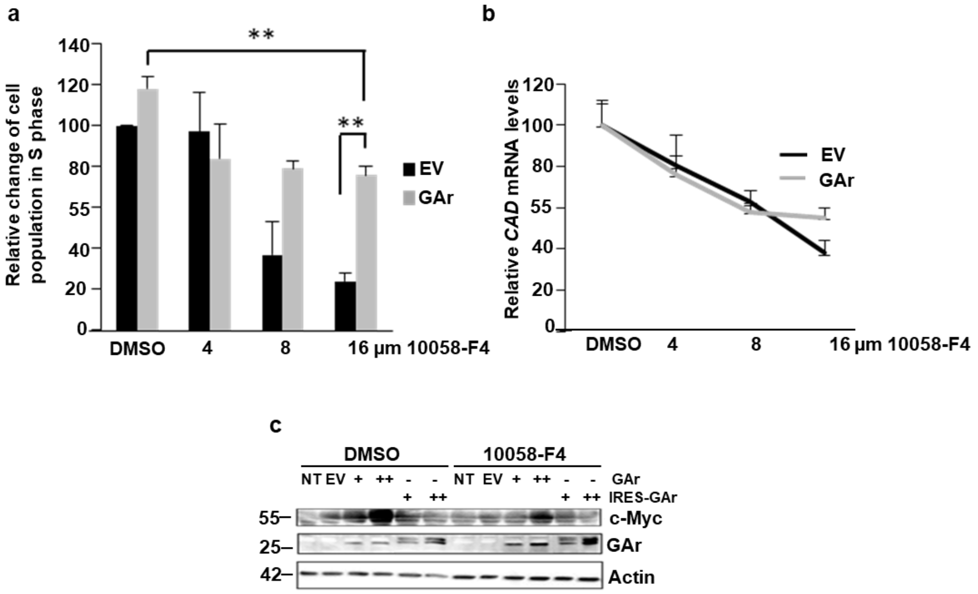

Supplementary Figure 3. a). FACS analysis of replicating cells following 48 hours of GAr expression, or control (EV), and treatment with increasing concentrations of the c-Myc inhibitor F4-10058 or DMSO control (16 hours before harvest). The data show relative values of cells in S-phase and the DMSO treated vector control is set to 100 %. b) Like in a), but RT-qPCR data show the relative levels of *CAD* mRNA. c) The presence of the 10058-F4 compound does not affect GAr-mediated induction c-Myc. Representation of mean data from three independent experiments with s.d. ($p^{**}<0.05$). Data relates to figure 1.

Supplementary Figure 4

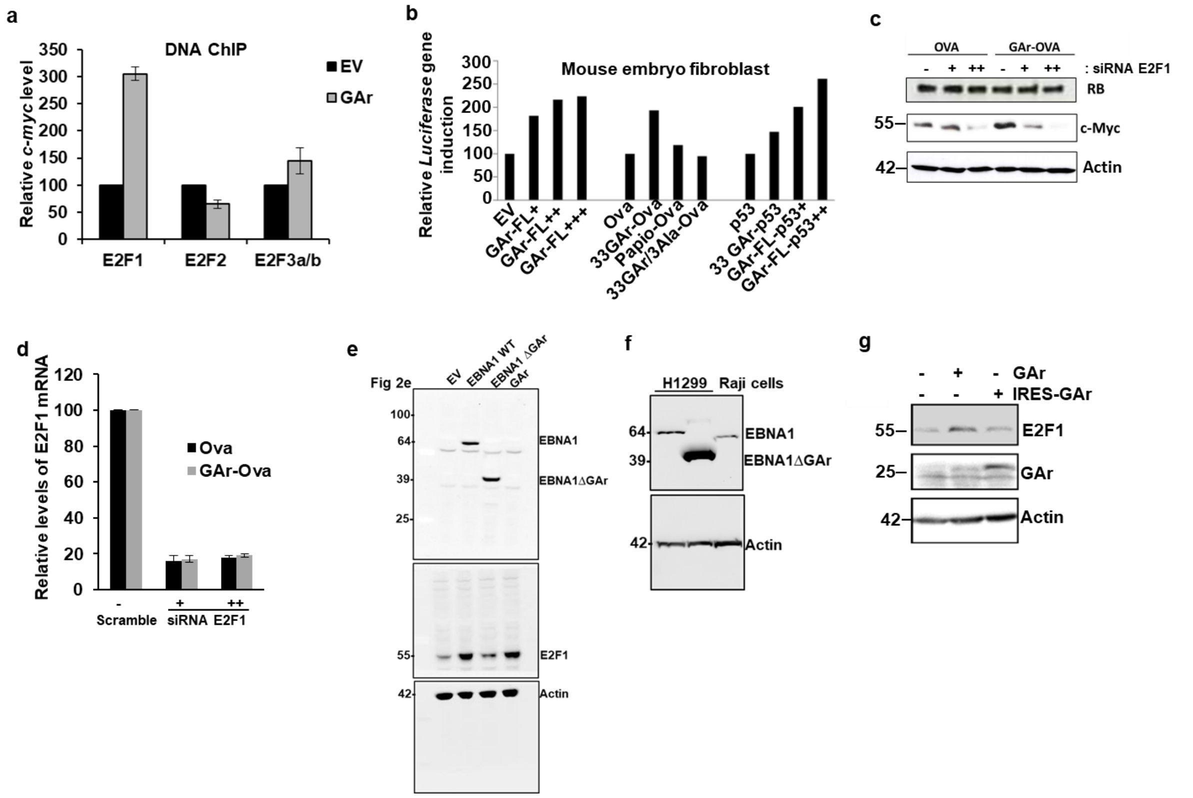

Supplementary Figure 4. a) Data from DNA ChIP assays using the (-83/+30) sequence of the endogenous *c-myc* promoter construct and antibodies against E2F1, E2F2 or E2F3a/b from cells expressing the GAr or EV. b) The effect of different GAr-carrying mRNAs on the human *myc*-promoter (-107/+34) fused to *luciferase* reporter gene in MEF cells. The Papio GAr has a serine residue inserted in every 8 codon and does not suppress mRNA translation. 33GAr-ova has a 33 long GAr fused in front of Ovalbumin. The 33GAr/3Ala has a disrupted GAr repeat sequence and is less effective in suppressing mRNA translation [1]. c) Silencing of *E2F1* mRNA affects c-Myc induction both in control (Ova) and GAr (GAr-Ova) transfected cells. d) Relative *E2F1* mRNA levels in Ova and GAr-Ova expressing cells (normalized against actin) treated with scramble siRNA and siRNA targeting E2F1. e) The full blot of main figure 2e as indicated. f) Comparison of EBNA1 protein levels between H1299 cells transfected with EBNA1 WT and EBNA1ΔGAr and Raji cells expressing endogenous EBNA1. g) Preventing the translation inhibitory activity of the GAr by inserting the *c-myc* IRES in the 5' UTR of the GAr-expressing mRNA abrogates induction of E2F1. The values in panels a and d represent the mean data from three independent experiments with s.d. Data relates to figure 2.

Supplementary Figure 5

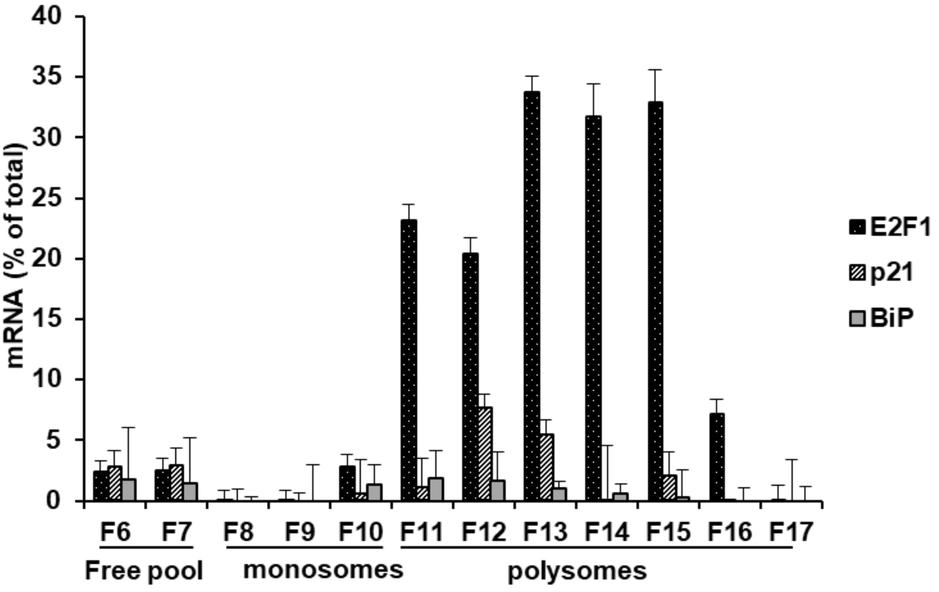

Supplementary Figure 5. Polysome profiling and RT-qPCR analysis of *E2F1, p21 and BiP* mRNAs in GAr expressing H1299 cells. The mRNA levels were normalised with actin in all fractions. The % of mRNA in each fraction from total mRNA was plotted. E2F1 mRNA levels show significant increase in polysomal fractions following GAr induction. The values represent the mean data of three independent experiments with s.d. Data relates to Figure 4c.

Supplementary Figure 6

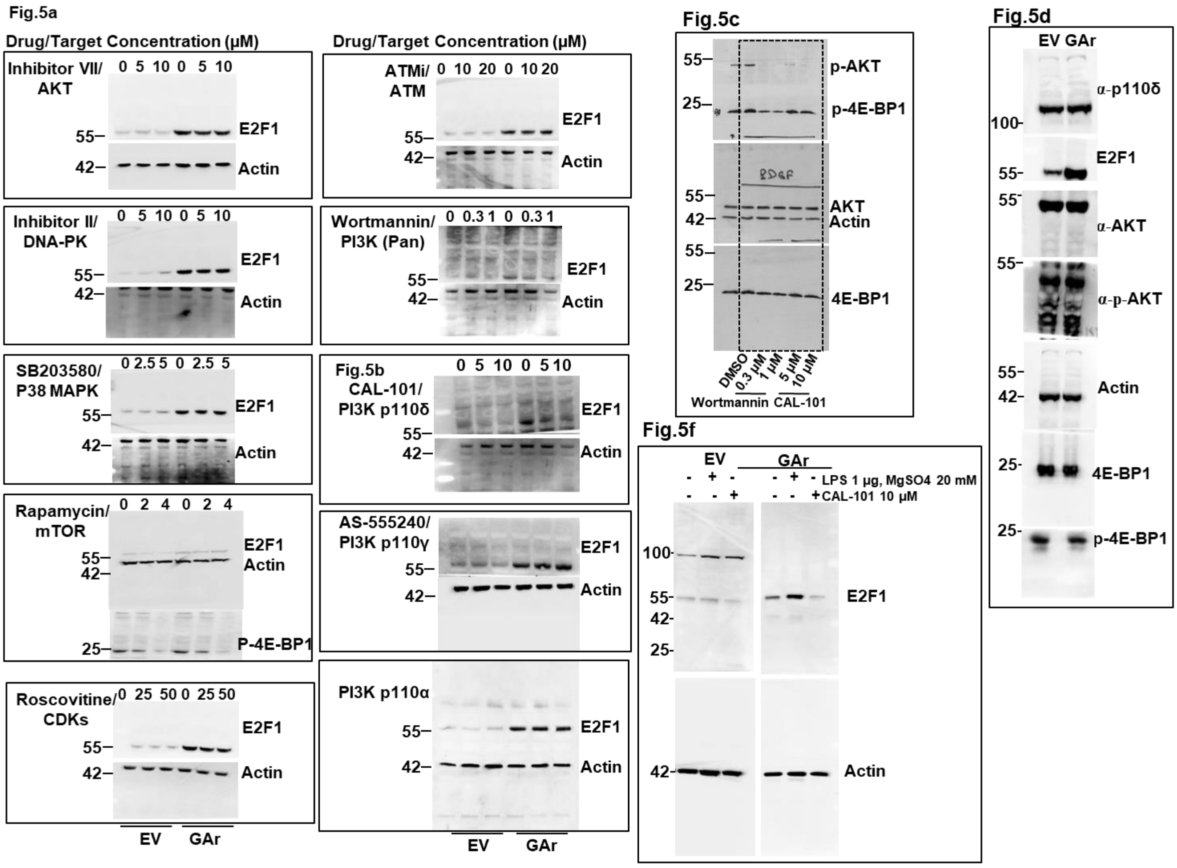

Supplementary Figure 6. Uncropped blots of main figures 5a, 5b, 5c, 5d and 5f as indicated.

Supplementary Figure 7

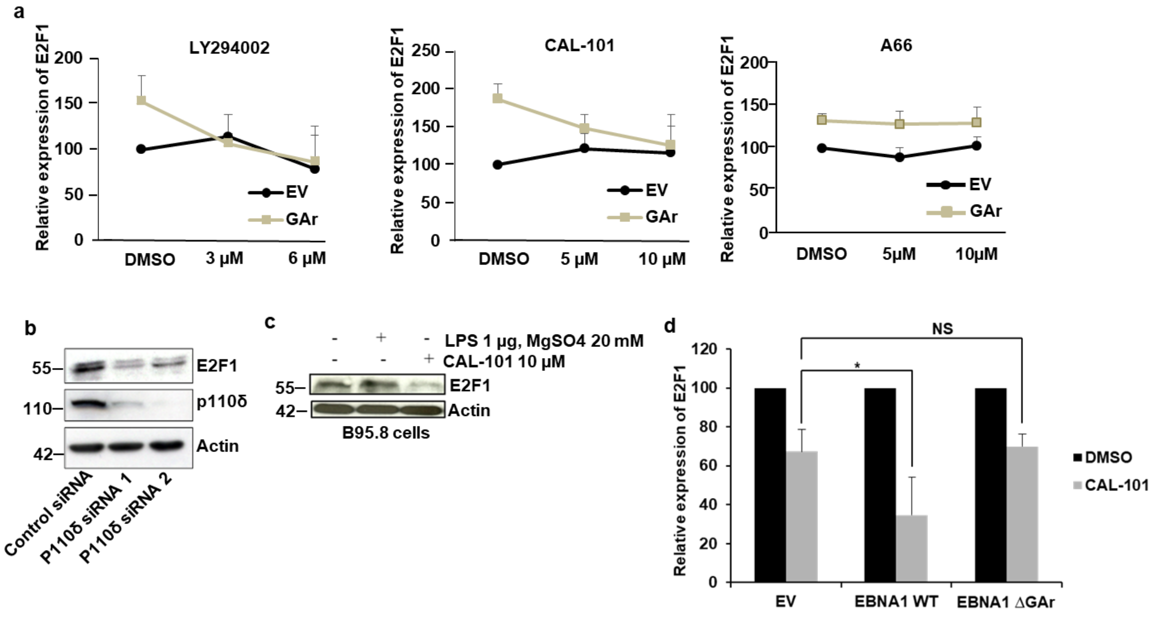

Supplementary Figure 7. a) The graphs show the relative mean values on E2F1 expression in H1299 cells treated with indicated drugs. b) Treatment of H1299 cells with two different siRNAs against p110δ suppresses the expression of E2F1. c) Western blot showing the expression of E2F1 proteins in EBV transformed B-lymphoblastoid cell line (B95.8) following induction of PI3Kδ with $MgSO_4$ (20 mM), LPS (1µg) and functional inactivation of PI3Kδ by treating with CAL-101. d) The graph shows CAL-101 (10 µM)-mediated suppression of E2F1 expression in H1299 cells expressing vector control (EV), EBNA1 WT or EBNA1 ∆GAr. The data shows that the relative suppression of E2F1 expression by CAL-101 is similar in control and EBNA1 ∆GAr-expressing cells and that the strongest effect is observed in cells expressing EBNA1 WT. The values represent the mean data from three independent experiments with s.d. Statistical significance was calculated using *t*-tests ($p^* < 0.1$ and NS- not significant). Data related to Figures 5 and 6.

Supplementary Figure 8

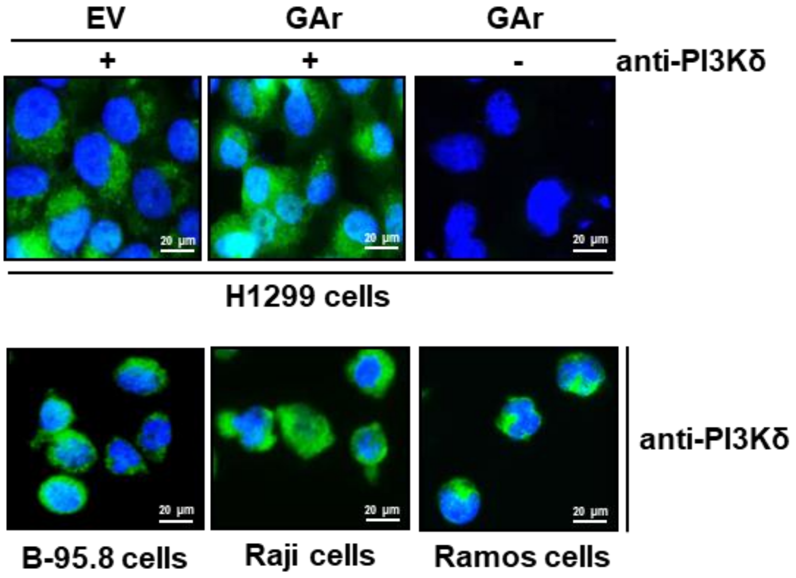

Supplementary Figure 8. Assessment of PI3Kδ localization upon GAr expression. Immunofluorescence staining was performed with PI3Kδ mouse monoclonal antibody (sc-136032) and goat anti-mouse antibody (Alexa flour 488, ab150113) and DAPI. The upper panel shows localization of PI3Kδ in H1299 cells expressing EV and GAr, cells expressing GAr probed without PI3Kδ antibody used as a negative control. The lower panel shows the localization of PI3Kδ in the EBV positive B95.8, Raji and the EBV negative Ramos cells. Data relates to figure 5.

Supplementary Figure 9

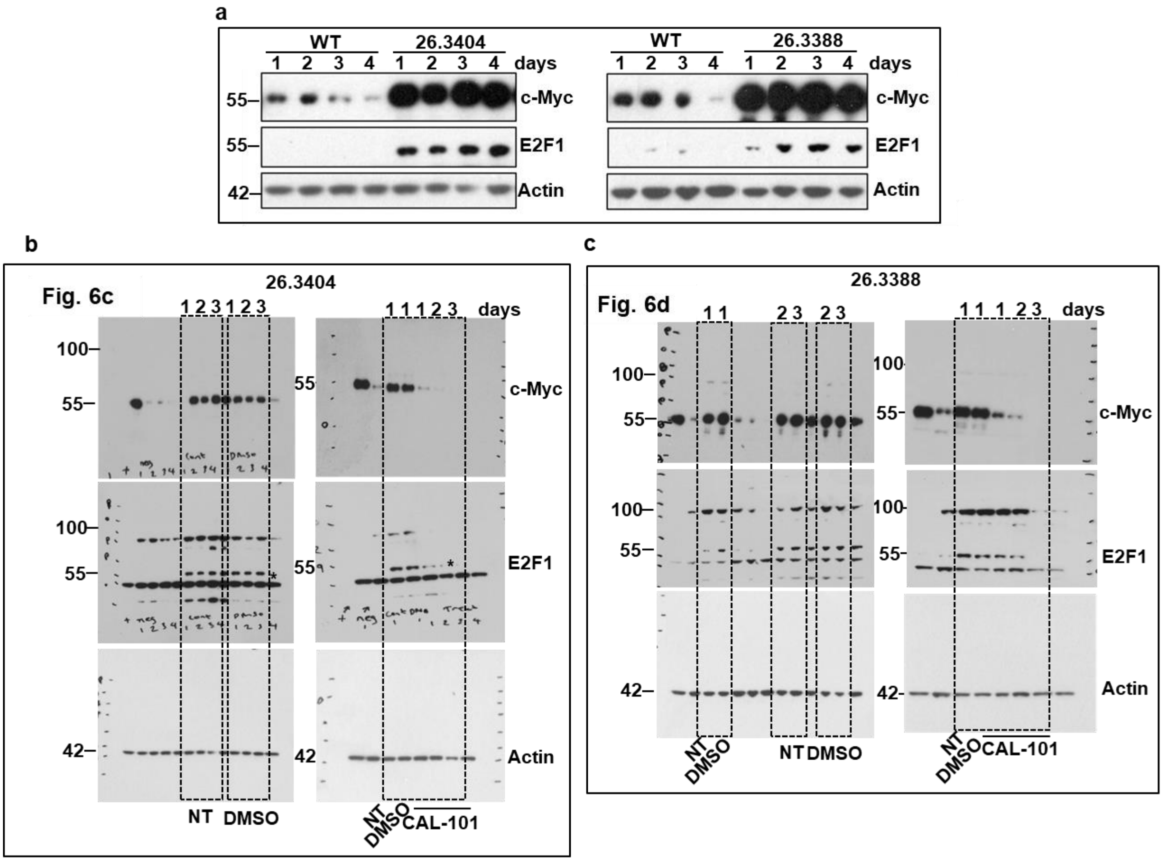

Supplementary Figure 9. a) Western blots showing a time course of the expression of E2F1 and c-Myc in non-transgenic wild type (WT) lymphocytes and in EBNA1 transgenic cells from two different primary tumours (ID:26.3404 and 26.3388) both were cultured for 4 days without any treatment, actin was used as a loading control. b) and c) Uncropped blots of main figures 6c and 6d respectively as indicated.

Supplementary Table. 1

Primers list.

Primer	Sequence 5'-3'	description
**Oligo**	**Sequence 5'-3'**	**description**
E2F1 CDS_f	GTGAGCGTCATGGCCTTG	forward primer for generating E2F1 CDS
E2F1 CDS_r	TCAGAAATCCAGGGGGGTGAGGTC	reverse primer for generating E2F1 CDS
E2F1_T1_f_HindIII	TATAAGCTTATGGACCTGCTGCTCTTCGCCACACCGCA	forward primer for generating E2F1 Δ(+1 to + 249)
E2F1_T2_f_HindIII	TATAAGCTTATGCGGGGCAGAGGCCGCCATCCAGGAA	forward primer for generating E2F1 Δ(+1 to + 324)
E2F1_T3_f_HindIII	TATAAGCTTATGCTGAACTGGGCTGCCGAGGT	forward primer for generating E2F1 Δ(+1 to + 432)
E2F1_T4_f_HindIII	TATAAGCTTATGGAGGGCATCCAGCTCATTGCCA	forward primer for generating E2F1 Δ(+1 to + 525)
E2F1_T5_f_HindIII	TATAAGCTTATGACCACAGTGGGCGTCGGCGGA	forward primer for generating E2F1 Δ(+1 to + 585)
E2F1_r_XhoI	TATCTCGAGTCAGAAATCCAGGGGGGTGAGGT	reverse primer for generating deletion clones of E2F1
E2F1_f_RT-qPCR	ACTCCTCGCAGATCGTCATCATCT	forward primer for E2F1 RT-qPCR
E2F1_r_RT-qPCR	GGACGTTGGTGATGTCATAGATGCG	reverse primer for E2F1 RT-qPCR
c-Myc (-83/+30)_f	AGGGCTTCTCAGAGGCTTG	forward primer for DNA-Chip assay
c-Myc (-83/+30) _r	TGCCTCTCGCTGGAATTACT	reverse primer for DNA-Chip assay
β-actin_qPCR_f	TCACCCACACTGTGCCCATCTACGA	forward primer for Actin RT-qPCR
βactin_qPCR_r	TGAGGTAGTCAGTCAGGTCCCG	reverse primer for Actin RT-qPCR
c-Myc-F	AACCAGAGTTTCATCTGCGACCCG	forward primer for c-Myc RT-qPCR
c-Myc-R	TTGTGCTGATGTGTGGAGACGTGG	reverse primer for c-Myc RT-qPCR
CAD-F	TGGGTGTGGACCTAGTAGCC	forward primer for CAD RT-qPCR
CAD-R	TGGGGATCTTAAAGCCAGTG	reverse primer for CAD RT-qPCR
cyclinE-F	GTTATAAGGGAGACGGGGAG	forward primer for CyclinE RT-qPCR
cyclinE-R	TGCTCTGCTTCTTACCGCTC	reverse primer for CyclinE RT-qPCR
cyclinB-F	TCTGGATAATGGTGAATGGACA	forward primer for Cyclin B RT-qPCR
cyclinB-R	CGATGTGGCATACTTGTTCTTG	reverse primer for Cyclin B RT-qPCR
GAPDH-F	GAGTCAACGGATTTGGTCGT	forward primer for GAPDH RT-qPCR
GAPDH-R	GACAAGCTTCCCGTTCTCAG	reverse primer for GAPDH RT-qPCR
Cyclin d1-F	TACTACCGCCTCACACGCTTC	forward primer for Cyclin d1 RT-qPCR
Cyclin d1-R	TTCGATCTGCTCCTGGCAG	reverse primer for Cyclin d1 RT-qPCR
CDK4-F	ATGTTGTCCGGCTGATGGA	forward primer for CDK4 RT-qPCR
CDK4-R	CACCAGGGTTACCTTGATCTCC	reverse primer for CDK4 RT-qPCR
RPL38-F	CGAAAGGATGCCAAATCTGTC	forward primer for RPL38 RT-qPCR
RPL38-R	TGCCTTCTCTTTGTCAGTGATG	reverse primer for RPL38 RT-qPCR
Rps19-F	CAG CAC GGC ACC TGT ACC T	forward primer for Rps19 RT-qPCR
Rps19-R	GCT GGG TCT GAC ACC GTT TC	reverse primer for Rps19 RT-qPCR
45S pre-rRNA-F	GCTGACACGCTGTCCTCTG	forward primer for 45S pre-rRNA-qPCR
45S pre-rRNA-R	ACGCGCGAGAGAACAGCAG	reverse primer for 45S pre-rRNA-qPCR
p21-F	CCTCAAATCGTCCAGCGACCTT	forward primer for p21-qPCR
p21-R	CATTGTGGGAGGAGCTGTGAAA	reverse primer for p21-qPCR
Bip-F	GCAACCAAAGACGCTGGAACT	forward primer for Bip-qPCR
Bip-R	CCTCCCTCTTATCCAGGCCATA	reverse primer for Bip-qPCR

Supplementary Reference

1. Apcher, S. *et al.* mRNA translation regulation by the Gly-Ala repeat of Epstein-Barr virus nuclear antigen 1. *Journal of virology* **83**, 1289-1298 (2009).
